# Identification of senescence-associated long non-coding RNAs to predict prognosis and immune microenvironment in patients with hepatocellular carcinoma

**DOI:** 10.3389/fgene.2022.956094

**Published:** 2022-10-13

**Authors:** Chengzhi Gao, Guangming Zhou, Min Cheng, Lan Feng, Pengbo Cao, Gangqiao Zhou

**Affiliations:** ^1^ State Key Laboratory of Proteomics, National Center for Protein Sciences at Beijing, Beijing Institute of Radiation Medicine, Beijing, China; ^2^ Collaborative Innovation Center for Personalized Cancer Medicine, Center for Global Health, School of Public Health, Nanjing Medical University, Nanjing, China; ^3^ Hebei University, Baoding, China; ^4^ Anhui Medical University, Hefei, China

**Keywords:** hepatocellular carcinoma, long noncoding RNA, prognostic signature, immune microenvironment, NRAV, oncogene-induced senescence

## Abstract

**Background:** Cellular senescence plays a complicated and vital role in cancer development because of its divergent effects on tumorigenicity. However, the long non-coding RNAs (lncRNAs) associated with tumor senescence and their prognostic value in hepatocellular carcinoma (HCC) remain unexplored.

**Methods:** The trans-cancer oncogene-induced senescence (OIS) signature was determined by gene set variation analysis (GSVA) in the cancer genome atlas (TCGA) dataset. The OIS-related lncRNAs were identified by correlation analyses. Cox regression analyses were used to screen lncRNAs associated with prognosis, and an optimal predictive model was created by regression analysis of the least absolute shrinkage and selection operator (LASSO). The performance of the model was evaluated by Kaplan-Meier survival analyses, nomograms, stratified survival analyses, and receiver operating characteristic curve (ROC) analyses. Gene set enrichment analysis (GSEA) and cell-type identification by estimating relative subsets of RNA transcripts (CIBERSORT) were carried out to explore the functional relevance and immune cell infiltration, respectively.

**Results:** Firstly, we examined the pan-cancer OIS signature, and found several types of cancer with OIS strongly associated with the survival of patients, including HCC. Subsequently, based on the OIS signature, we identified 76 OIS-related lncRNAs with prognostic values in HCC. We then established an optimal prognostic model based on 11 (including *NRAV, AC015908.3, MIR100HG, AL365203.2, AC009005.1, SNHG3, LINC01138, AC090192.2, AC008622.2, AL139423.1,* and *AC026356.1*) of these lncRNAs by LASSO-Cox regression analysis. It was then confirmed that the risk score was an independent and potential risk indicator for overall survival (OS) (HR [95% CI] = 4.90 [2.74–8.70], *p* < 0.001), which outperforms those traditional clinicopathological factors. Furthermore, patients with higher risk scores also showed more advanced levels of a proinflammatory senescence-associated secretory phenotype (SASP), higher infiltration of regulatory T (Treg) cells and lower infiltration of naïve B cells, suggesting the regulatory effects of OIS on immune microenvironment. Additionally, we identified *NRAV* as a representative OIS-related lncRNA, which is over-expressed in HCC tumors mainly driven by DNA hypomethylation.

**Conclusion:** Based on 11 OIS-related lncRNAs, we established a promising prognostic predictor for HCC patients, and highlighted the potential immune microenvironment-modulatory roles of OIS in HCC, providing a broad molecular perspective of tumor senescence.

## Introduction

Hepatocellular carcinoma (HCC) is the fourth leading cause of cancer-related mortality globally (Villanueva, 2019). Infection with chronic hepatitis B virus (HBV) is a key risk factor for HCC in developing nations such as China and several African countries (Ligat et al., 2019). Other risk factors for HCC include hepatitis C virus (HCV) infection, aflatoxin contact, alcohol use, smoking and diabetes (Jemal et al., 2011). Though the advances of modern medicine and the adoption of several treatment techniques have improved the outcomes of HCC patients, the long-term prognosis after surgical resection remains restricted due to the high risk of metastasis or recurrence (Wang et al., 2012).

Prognostic biomarkers are useful for assessing survival, determining therapy strategies and selecting individuals who are suitable candidates for clinical trials (Epstein et al., 2016). Biomarkers like tumor size, lymph node count, and metastasis status have historically been used to predict prognosis in oncology. However, the accuracies of these clinicopathologic variables are restricted (Tseng et al., 2014). Thus, molecular biomarkers are increasingly being used instead of, or in addition to, these clinicopathologic characteristics. In the case of HCC, serum α-fetoprotein (AFP), AFP-L3 and des-γ-carboxyprothrombin are being utilized or studied for the early detection and monitoring of HCC patients (Ocker, 2018). However, little progress has been made in the development of prognostic biomarkers for reliable HCC survival assessment and therapeutic approach decision (Black and Mehta, 2018).

Cellular senescence is a permanent condition of cell growth arrest that can promote tissue remodeling or contribute to inflammation as one of the major intrinsic fail-safe mechanisms. Because of the crucial roles it plays in tumorigenesis, the characteristics of senescent cells including identification and pharmacological elimination, have gained great attention in the field of tumor research. In addition to the replicative senescence induced by alteration of the telomere, oncogene-induced senescence (OIS) is another type of cellular senescence (Serrano et al., 1997). Activation or over-expression of oncogenes associated with OIS was once considered to provide a barrier against tumor development by triggering a series of molecular and cellular changes that eventually lead to cell cycle arrest. However, senescent cells can also gain a phenotype that increases the level of cytokines, chemokines, matrix metalloproteinases (MMPs), and other proteins in local environment, termed as senescence-associated secretory phenotype (SASP) (Coppé et al., 2010). Senescent cells with SASP can have a great influence on the immune microenvironment of tumor and render it to a conducive status to tumor growth and progression (Park et al., 2021). As there still remains a paradox about the roles senescence plays in cancer development and the immune microenvironment, the molecular landmarks of tumor senescence that may help elucidate tumor initiation and progression are worth investigating.

Although some studies indicate that prohibiting *p16*, *p21,* and *p53* accumulation can change the status of cellular senescence (Jung et al., 2016), the exact regulatory mechanism of cellular senescence is still largely unknown. It has recently emerged that lncRNAs can play important regulatory roles (Ghanam et al., 2017) and a few of them have been demonstrated linked to senescence as key players during direct/indirect regulation of oncogenes and SASP induction. *PANDA*, a lncRNA induced by DNA damage, might, for example, control senescence by blocking transactivation of proliferative genes associated with senescence (Puvvula et al., 2014). The lncRNA *MIR31HG* regulates BRAF-induced senescence by affecting the expression and secretion of some SASP components (Montes et al., 2021). Besides, downregulating lncRNA *MALAT* can influence p*53* activation in cycling cells, which can also induce senescence (Tripathi et al., 2013). These findings emphasized the importance of lncRNAs as regulators in cellular senescence and emphasized the need for more in-depth studies of senescence-related lncRNAs. Meanwhile, lncRNAs have been found to be closely related to tumor development, metastasis, and outcomes in HCC patients (Huang et al., 2020). However, studies to date are still unable to identify the senescence-related lncRNAs that can be regarded as molecular landmarks and hopeful prognostic predictors in HCC.

Here, we leveraged the multi-omics datasets of the cancer genome atlas (TCGA) and explored the prognostic value of senescence-related lncRNAs. Several senescence-related lncRNAs were identified as predictive prognostic biomarkers, using which a prognosis model was constructed for HCC patients. The model was further refined and confirmed by comprehensive assessment and *NRAV* was prioritized as a potential novel regulator of OIS. Moreover, we profiled an OIS-related abnormal developmental process which can reflect the oncogenic pattern of SASP in HCC. We also found that the senescent cells with advanced levels of OIS can possibly render the immune microenvironment to a conducive status to tumor growth and progression with more abundant regulatory T cells and less naïve B cells infiltration.

## Materials and methods

### Data collection

Raw count data of RNA sequencing in HCC tumors and non-tumor liver tissues was accessed through the TCGA data portal (https://gdc-portal.nci.nih.gov/). Pan-cancer multi-omics data and clinical data were obtained from the TCGA Pan-Cancer Atlas (https://gdc.cancer.gov/about-data/publications/pancanatlas) and GEPIA (Tang et al., 2017), respectively. TCGA liver hepatocellular carcinoma (TCGA-LIHC) dataset (including 374 HCC tumor tissues and 50 adjacent non-tumor tissues) was used for the identification of differently expressed genes. Patients with OS less than 30 days were excluded to remove potential bias related to treatment effects and a total of 346 HCC tumor samples were finally kept to develop the prognostic risk model. GSE144269 (Candia et al., 2020) (including 70 HCC tumor tissues with survival information) available in the Gene Expression Omnibus (GEO: https://www.ncbi.nlm.nih.gov/geo) database was obtained to evaluate the prediction ability of the model. The information of all datasets was shown in Supplementary Table S1.

### Identification of senescence-related lncRNAs

A total of 2,365 OIS-related genes were collected from the previous study (Hernandez-Segura et al., 2017). Among them, 1,219 genes are up-regulated and the rest 1,146 genes are down-regulated along the OIS process. The gene set variation analysis (GSVA) package (Hänzelmann et al., 2013) was used to compute OIS gene set scores: OIS score = (GSVA score of the OIS-up-regulated genes in OIS)–(GSVA score of the OIS-down-regulated genes). Candidate lncRNAs were then selected according to the correlation between the OIS scores and the lncRNA expression levels (Spearman rank correlation; *p* < 0.05; abs [correlation coefficient *rho*] ≥ 0.4).

### Construction and evaluation of the prognostic risk model for hepatocellular carcinoma patients

For the building of the prognostic risk score model, we employed a two-stage procedure. First, for each candidate lncRNA, univariate Cox proportional hazards regression analysis was performed to identify the lncRNAs correlated with HCC OS in the model training cohort. Prognosis-related lncRNAs were defined as those with a *p* value less than 0.05. Then, using the GLMNET package (Friedman et al., 2010) in R, we developed models with least absolute shrinkage and selection operator (LASSO) regression analysis. LASSO is a method for shrinking regression coefficients based on a penalization factor (lambda). Some coefficients may be shrunk to zero and hence eliminated from the model. The LASSO regression with Cox proportional hazards model was used in the model training cohort, and the optimal lambda was determined based on a 10-fold cross validation. The coefficients were then estimated. If the coefficient was zero, the lncRNAs were deleted, and the remaining lncRNAs were utilized to build the prognostic model. Based on the coefficients and the expression level of each candidate lncRNA, the following algorithm was used to calculate the risk score for each HCC case:

$$Risk\;score=\overset{n}{\underset{i=1}{\sum }}Coef\left({lncRNA}_{i}\right)*Expr\left({lncRNA}_{i}\right)$$

The median risk score was used to categorize HCC patients as “high-risk” or “low-risk”. Using the rms package (Harrell, 2015) in R, a nomogram was created from the model to analyze the OS of HCC patients. The suitability of the model to the nomogram was then evaluated by 1-, 3-, and 5-year calibration curves. Besides, the area under the receiver operating characteristic (ROC) curve was also employed to assess the regression model’s prediction performance.

### Functional enrichment analysis

The clusterProfiler package (Wu et al., 2021) was used to perform gene ontology (GO) and KEGG pathway enrichment analyses, and the enrichplot function was used to show the connection between the enriched pathways. Gene Set Enrichment Analysis (GSEA) (Subramanian et al., 2005) was performed by GSEA software (v 4.1.0) using all gene sets documented in the MSigDB-C5 (v 7.3). The gene set with an absolute normalized enrichment score (NES) > 1 and a false discovery rate (FDR, *q*) less than 0.05 was considered statistically significant. The SASP signature was collected from the previous study (Coppé et al., 2010) to compare the functional differences among risk groups.

### Estimation of the immune cells infiltration based on bulk RNA-seq data of hepatocellular carcinoma tumors

CIBERSORT (Newman et al., 2015) was used to quantify immune infiltration in tumor samples, which was performed in R using the IOBR package (Zeng et al., 2021) and set to absolute quantification output with 200 permutations. As input, gene-level transcripts per million (TPM) were employed.

### Statistical analysis

All statistical analyses were performed with R (v 4.1.0). Differential analysis of RNA-seq read counts was performed by using the DESeq2 package (Love et al., 2014). For survival analyses, Kaplan-Meier survival curves were generated using the survival R package and log-rank test was used to compare the difference in survival curves between two groups. Using univariate Cox proportional hazards regression analysis, the HR and 95 percent confidence interval (CI) were computed. *p* < 0.05 were considered statistically significant in all statistical tests. visualization was done with the ggplot2 (Wickham, 2016) or ggpubr (Kassambara and Kassambara, 2020) R packages. Principal component analysis (PCA) was applied in the visualization of high-dimensional data and the result was plotted with scatterplot3d (Ligges and Maechler, 2003).

## Results

### Pan-cancer prognostic evaluation of the oncogene-induced senescence signature and identification of the oncogene-induced senescence-related lncRNAs in hepatocellular carcinoma

**FIGURE 1:**
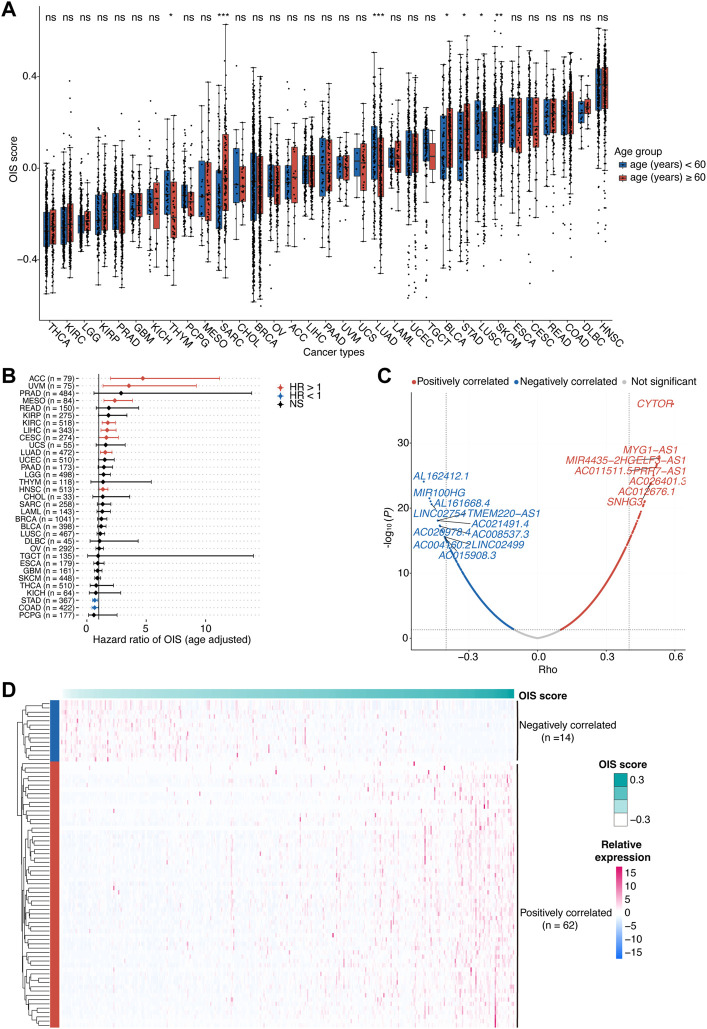
Identification of OIS-related lncRNAs in HCC.**(A)** Oncogene-induced senescence (OIS) scores of tumor tissues across cancer types from TCGA. Wilcox test was used to assess the difference across different age groups. **(B)** The correlation between OIS score and OS of patients across all cancer types in TCGA. The HRs and 95% CIs were identified by multivariate Cox proportional hazards analyses. **(C)** A volcano plot of the correlation between expression levels of lncRNAs and OIS scores in TCGA-LIHC patients. The *red spots* represent the statistically significant positive correlates and the *blue* ones represent the statistically significant negative correlates. The correlation coefficients (*rho*) and *p* values were determined by Spearman rank correlation analysis. **(D)** An expression heatmap of OIS-related lncRNAs in HCC tumors from TCGA. OIS, oncogene-induced senescence; HCC, hepatocellular carcinoma; TCGA, the cancer genome atlas; OS, overall survival; HR, hazards ratio; CI, confidence interval.

Cellular senescence has been reported in several studies to affect the prognosis of patients with cancer, but the pan-cancer features of OIS have not been well elucidated. We collected an OIS-associated gene expression signature from a previous study (Hernandez-Segura et al., 2017), including a total of 2,365 protein-coding genes. To evaluate the association of cellular senescence with the prognosis of cancer patients, we calculated the OIS score using GSVA across all 33 types of cancer in TCGA (Figure 1A). We observed distinct OIS scores in different types of cancer, higher in head and neck squamous cell carcinoma (HNSC), colon adenocarcinoma (COAD) and lymphoid neoplasm diffuse large B-cell lymphoma (DLBC), while lower in kidney renal clear cell carcinoma (KIRC), thyroid carcinoma (THCA) and brain lower grade glioma (LGG). Of note, the OIS scores were moderately correlated with the ages of patients in 7 out of the 33 cancer types, suggesting certain effects of aging on the OIS signature. To eliminate the influence of this covariant, all subsequent analyses were performed after correction for age.

Next, we examined the association of the OIS score with the prognosis of patients. Using the median OIS score, patients with each type of cancer were divided into groups with higher and lower OIS scores, and the survival analyses were performed by Univariant Cox proportional hazards regression analyses. It showed that the OIS scores of patients in 10 different types of cancer were significantly correlated with OS (Figure 1B). Among them, OIS scores indicated decreased risks of death (HR < 1) in stomach adenocarcinoma (STAD) and colon adenocarcinoma (COAD), while indicated increased risks of death (HR > 1) in the other eight types of cancer, including adrenocortical carcinoma (ACC), uveal melanoma (UVM), mesothelioma (MESO), kidney renal clear cell carcinoma (KIRC), LIHC, lung adenocarcinoma (LUAD), cervical squamous cell carcinoma and endocervical adenocarcinoma (CESC), head and neck squamous cell carcinoma (HNSC), suggesting the adverse effects of OIS in cancer progression.

There is considerable evidence that lncRNAs provide an additional degree of complexity to the regulation of genes, including several famous OIS-related genes like *INK4A* (Montes et al., 2015), *KRAS* (La Montagna et al., 2021), *NRAS* (Chen et al., 2021) and *PTEN* (Zang et al., 2020). Given the prognostic risk of OIS in HCC and the pivotal roles of lncRNAs in cellular senescence (Abdelmohsen and Gorospe, 2015), we therefore went on to identify the OIS-related lncRNAs in HCC. Through Spearman rank correlation analyses, we finally identified a total of 76 lncRNAs with their expression levels significantly correlated with OIS scores in HCC tumors (abs [*rho*] > 0.4 and *p <* 0.05; Figure 1C), including 62 positively correlated and 14 negatively correlated ones (Figure 1D). Furthermore, many of the OIS-related lncRNAs we identified have been demonstrated as pivotal contributors to cellular senescence. For example, *SNHG3* (*rho* = 0.50, *p* < 0.01) can act as a ceRNA for *miR-485* to up-regulate *ATG7* expression (Cao et al., 2020); *MIR100HG* (*rho* = -0.47, *p* < 0.01) has the ability to epigenetically silence *LATS1* and *LATS2* (Su et al., 2019); and *ZFAS1* (*rho* = 0.43, *p* < 0.01) can influence the regulatory axis of *miR-373–3 p/MMP3* (Wang G. et al., 2021). Except for the impact on these OIS-related genes, some lncRNAs can also participate in the process of senescence directly, like *PVT1* (*rho* = 0.41, *p* < 0.01) can inhibit tendon stem/progenitor cell senescence by sponging *miRNA-199a-5p* (Han et al., 2022); and *SNHG12* (*rho* = 0.44, *p* < 0.01) can interact with DNA-dependent protein kinase and protect vascular endothelium from senescence (Haemmig et al., 2020). Together, we found the heterogeneous prognostic value of OIS across different types of cancer and highlighted the diverse effects of OIS in HCC.

### Construction of prognostic risk score model for hepatocellular carcinoma

**FIGURE 2:**
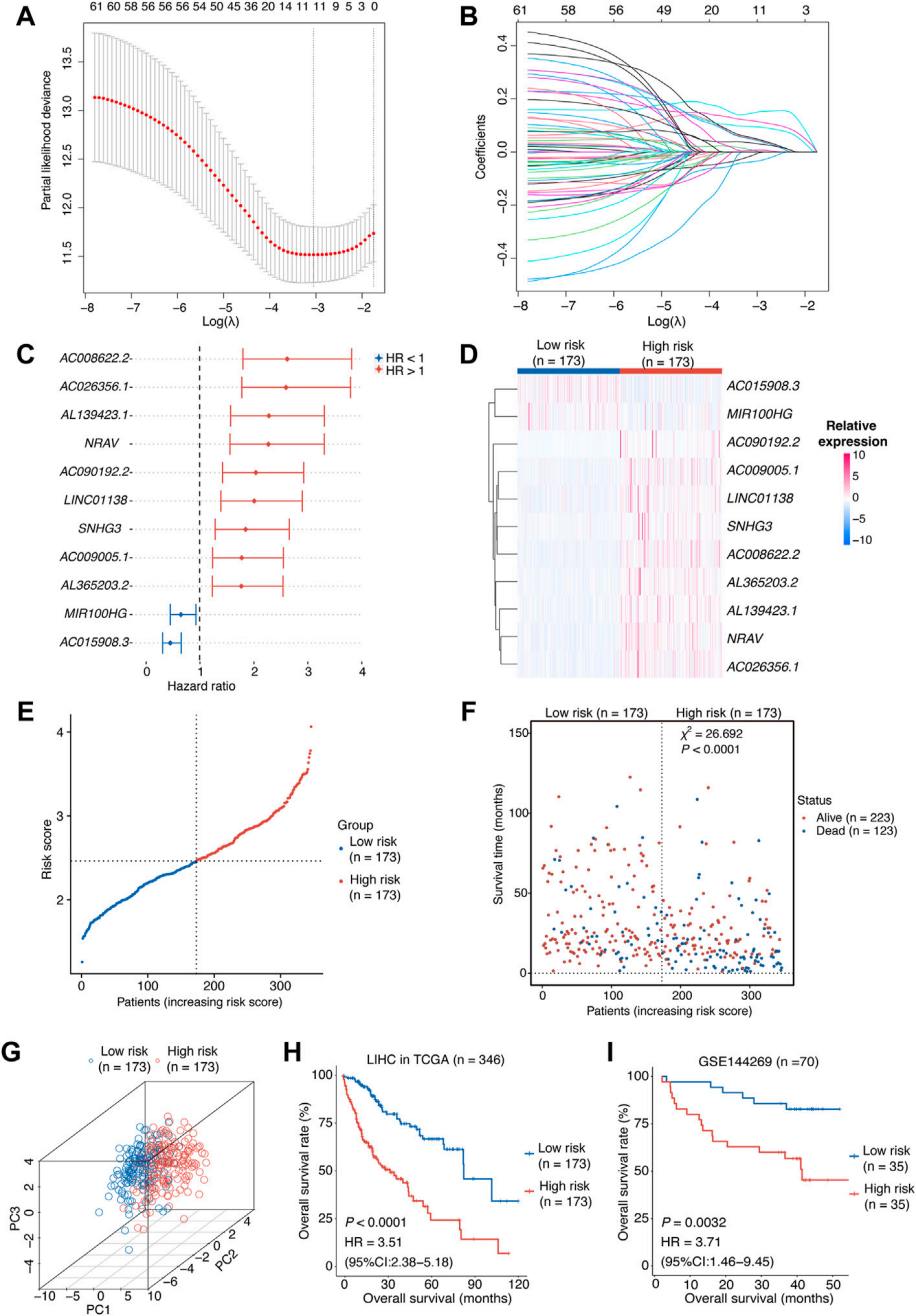
Construction of the risk score model.**(A,B)** OIS-related lncRNA candidates for constructing the prognostic model (obtained 11 non-zero coefficients from LASSO-Cox regression analysis). **(C)** The HRs and 95% CIs of the 11 optimal OIS-related prognostic lncRNAs identified by Cox regression analysis. **(D)** A heatmap of expression levels of the 11 lncRNAs in the risk score model. **(E)** The rank of HCC patients according to the risk score. **(F)** The correlation between the HCC patients’ prognosis and the risk score. The *p* value was computed using *χ* ^2^ test. **(G)** A three-dimensional scatter PCA plot of HCC patients according to the risk score. **(H)** Kaplan-Meier survival plots of HCC patients from the training cohort (HCC tumors from TCGA) with a high or low risk score according to the 11-OIS-related-lncRNA prognostic risk model. **(I)** Kaplan-Meier survival plots of HCC patients from the validation cohort (GSE144269) with a high or low risk score according to the 11-OIS-related-lncRNA prognostic risk model. OIS, oncogene-induced senescence; HR, hazards ratio; CI, confidence interval; HCC, hepatocellular carcinoma; PCA, principal component analysis; TCGA, the cancer genome atlas.

To further identify prognosis-associated lncRNAs among these 76 candidates in HCCs, we initially assessed associations between these candidate lncRNAs and clinical outcomes by utilizing the univariant Cox proportional hazards regression analysis in the training cohort (TCGA-LIHC), after which 62 ones survived. Next, we employed a LASSO-Cox regression model to construct a prognostic classifier, from which we chose 11 optimum lncRNAs (Figures 2A,B), including nine risk lncRNAs (*NRAV*, *AL365203.2*, *AC009005.1*, *SNHG3*, *LINC01138*, *AC090192.2*, *AC008622.2*, *AL139423.1,* and *AC026356.1*) with HR > 1 and two protective lncRNAs (*MIR100HG* and *AC015908.3*) with HR < 1 (Figure 2C).

The risk score for each HCC case was computed using the expression levels of these 11 lncRNAs and their LASSO regression coefficients: Risk score = 0.1483 × *NRAV* + 0.1160 × *AL1*39423.1 + 0.0950×*AC*008622.2 + 0.0513 × *AL*365203.2 + 0.0485 × *LINC*01138 + 0.0453 × *AC*009005.1 + 0.0326 × *AC*090192.2 + 0.0069 × *SNH*G3 + 0.0051 × *AC*026356.1 − 0.0094 × *MIR*100HG − 0.0355 × *AC*015908.3 According to the median risk score, we grouped the TCGA-LIHC cohort patients into two groups: high-risk and low-risk. The expression levels of these 11 lncRNAs were consistent with the risk group we defined (Figure 2D). The risk score can be attached to the status of survival because patients in the high-risk group tend to have worse prognosis (Figures 2E,F). Meanwhile, the principal component analysis (PCA) indicated that these lncRNAs were capable of distinguishing the high-risk group from the low-risk group (Figure 2G). Survival analyses in the TCGA-LIHC cohort revealed that the individuals with higher risk scores displayed a shorter OS than those with lower risk scores (*p* < 0.0001, HR [95% CI] = 3.51 [2.38–5.18]; Figure 2H). We tested our model in an independent HCC cohort (GSE144269) (Candia et al., 2020), which contains 70 HCC tumor tissues with survival information, to confirm its predictive value. As expected, the risk score was significantly associated with the OS of HCC patients (*p =* 0.0032, HR [95% CI] = 3.71 [1.46–9.45]; Figure 2I). Taken together, we constructed an 11-OIS-related-lncRNA prognostic model for patients with HCC.

### Comprehensive assessment of the prognostic model

**FIGURE 3:**
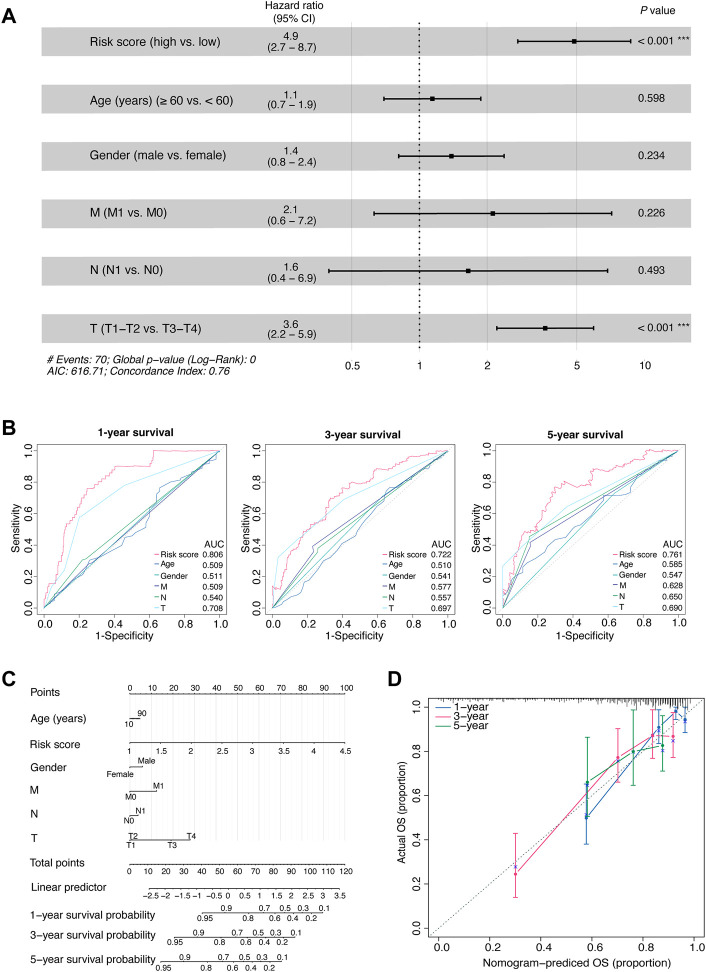
Comprehensive assessment of the prognostic model.**(A)** The HRs with 95% CIs, and *p* values of the risk score and clinical factors determined by multivariate Cox regression analyses. **(B)** Time-dependent AUCs of the risk score and clinical factors based on the ROC curves for predicting 1-, 3-, and 5-year survival (including age, gender, M stage, N stage, and T stage). **(C)** An integrated nomogram for predicting the possibilities of 1-year, 3-year and 5-year survival for TCGA-LIHC cohort. The prognostic nomogram was constructed using the risk score from the 11-OIS-related-lncRNA prognostic risk model and clinical factors. **(D)** Calibration curves of the concordance between the predicted and observed 1-, 3-, and 5-year survival rates of HCC patients from the TCGA cohort based on the prognostic nomogram. Nomogram-predicted OS is plotted on the x-axis; the actual OS is plotted on the y-axis. The diagonal dotted line represents a perfect prediction by an ideal model. OIS, oncogene-induced senescence; HR, hazards ratio; CI, confidence interval; AUC, area under the curve; ROC, receiver operating characteristic; T, tumor; N, node; M, metastasis; HCC, hepatocellular carcinoma; TCGA, the cancer genome atlas; OS, overall survival.

Next, we comprehensively assessed the prognostic model. First, we performed multivariate Cox proportional hazards regression analysis and found that the risk score, rather than most of the clinicopathological factors, exhibited independent prognostic value for patients with HCC (*p* < 0.001, HR [95% CI] = 4.9 [2.74–8.70]; Figure 3A). Subsequently, we tested the ability of the risk model to predict short-term and long-term viability of HCC patients and found that the 1-, 3-, and 5-year survival yielded AUC values of 0.806, 0.722, and 0.761, respectively (Figure 3B). Of note, the ability of the risk score to predict short-term or long-term survival outperformed the clinicopathological factors including age, gender and TNM stage (all AUCs ≤0.7).

Then, to develop a clinically quantitative method for predicting the probabilities of 1-, 3-, and 5-year OS in HCC patients, we performed multivariate Cox proportional hazards regression analyses and generated an integrated nomogram. The predictors were composed of the risk score and the clinicopathological factors (including age, gender, and TNM stage; Figure 3C). Next, the calibration curves showed that the predicted 1-, 3-, and 5-year survival probabilities matched the actual survival probabilities, respectively (Figure 3D).

**FIGURE 4:**
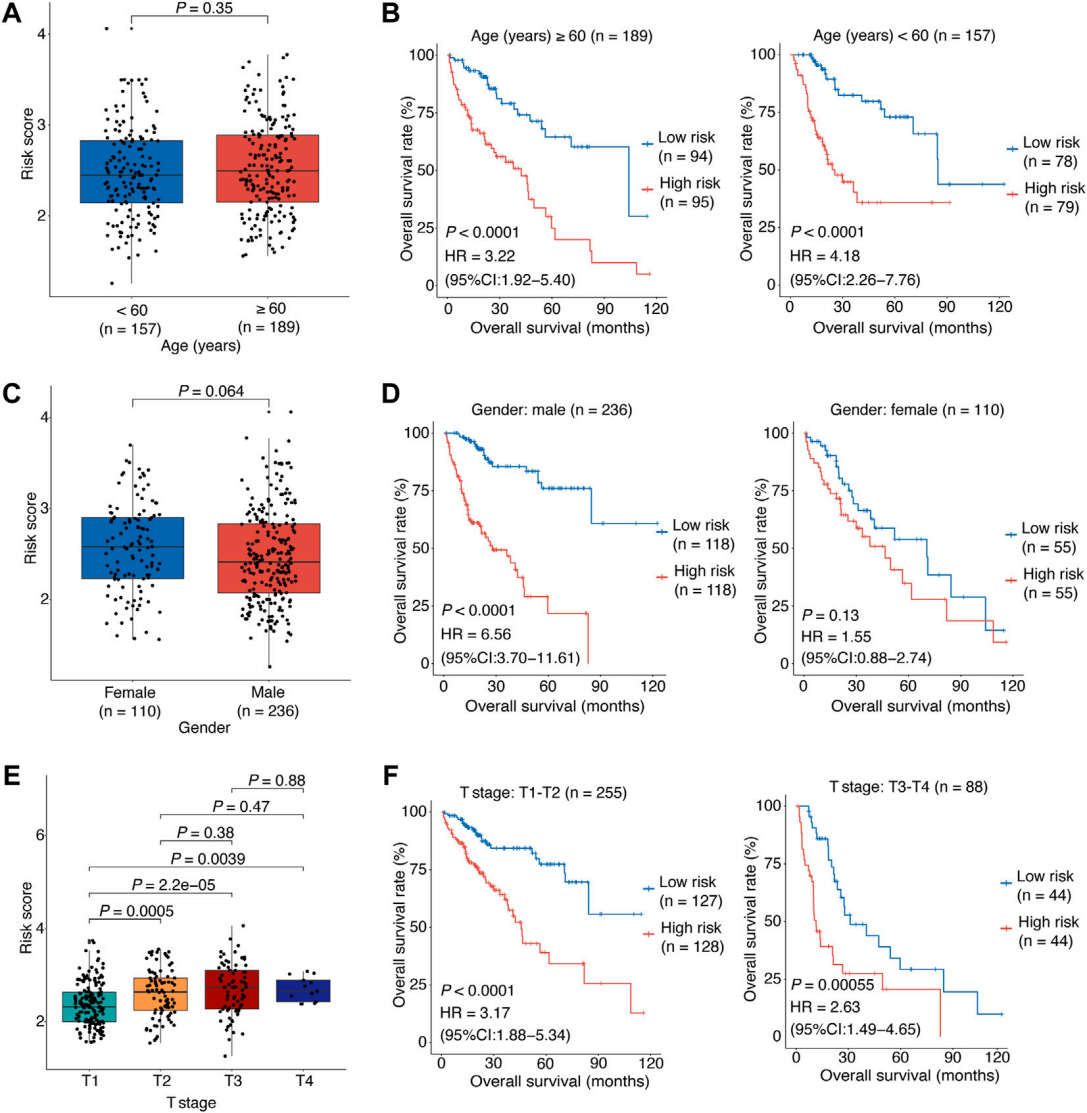
Stratified analyses of association between the risk score and HCC overall survival.**(A)** The correlation between the risk score and the patient’s age. **(B)** The risk score’s predictive value in HCC patients stratified by age (≥60 vs. < 60 years). **(C)** The correlation between the risk score and the patient’s gender. **(D)** The risk score’s predictive value in HCC patients stratified by gender (male vs. female). **(E)** The correlation between the risk score and the patient’s clinical stage. **(F)** The risk score’s predictive value in HCC patients stratified by clinical stage (T1-T2 vs. T3-T4). In **(A–E)**, the *p* values were determined by Wilcox test. In **(B–F)**, the patients were stratified by the median of the risk scores for each stratified group to perform Kaplan-Meier OS analysis; the *p* values were determined by log-rank test and the HRs with 95% CIs were obtained from multivariate Cox proportional hazards regression analyses. HCC, hepatocellular carcinoma; HR, hazards ratio; CI, confidence interval; OS, overall survival.

Finally, stratified survival analyses were performed to further evaluate the relationship between the risk score and clinical characteristic factors in HCC. Between the young and senior patients, the risk scores were statistically comparable (*p* = 0.35; Figure 4A), excluding the effect of aging on OIS in HCC. The Kaplan-Meier survival analyses revealed that the OS rate was considerably lower for the high-risk patients compared to the low-risk patients both in the old group (*p* < 0.0001, HR [95% CI] = 3.22 [1.92–5.4]; Figure 4A) and the young group (*p* < 0.0001, HR [95% CI] = 4.18 [2.26–7.76]; Figure 4A). We also observed no difference in risk scores between male and female patients (*p* = 0.064; Figure 4B). The risk model’s prognostic usefulness was seen in male patients (*p* < 0.0001, HR [95% CI] = 6.56 [3.7–11.61]; Figure 4B). For female patients, there was no evidence of a significant predictive value (*p* = 0.13, HR [95% CI] = 1.55 [0.88–2.74]; Figure 4B). Furthermore, HCC patients in higher clinical stages (T2, T3, and T4 stage) had higher risk scores than those in lower clinical stage (T1 stage; Figure 4C). The OS rate was significantly lower for the high-risk patients compared to the low-risk individuals in both early (T1-T2 stage; *p* < 0.0001, HR [95% CI] = 3.17 [1.88–5.34]; Figure 4C) and advanced stage patients (T3-T4 stage; *p* < 0.00055, HR [95% CI] = 2.63 [1.49–4.65]; Figure 4C).

Overall, the above results showed that our model had accurate and reliable performance in HCC OS prediction and the risk score is a significant independent prognostic factor, especially for male patients or those with early stage.

### Different biological implication of the oncogene-induced senescence-related risk score

**FIGURE 5:**
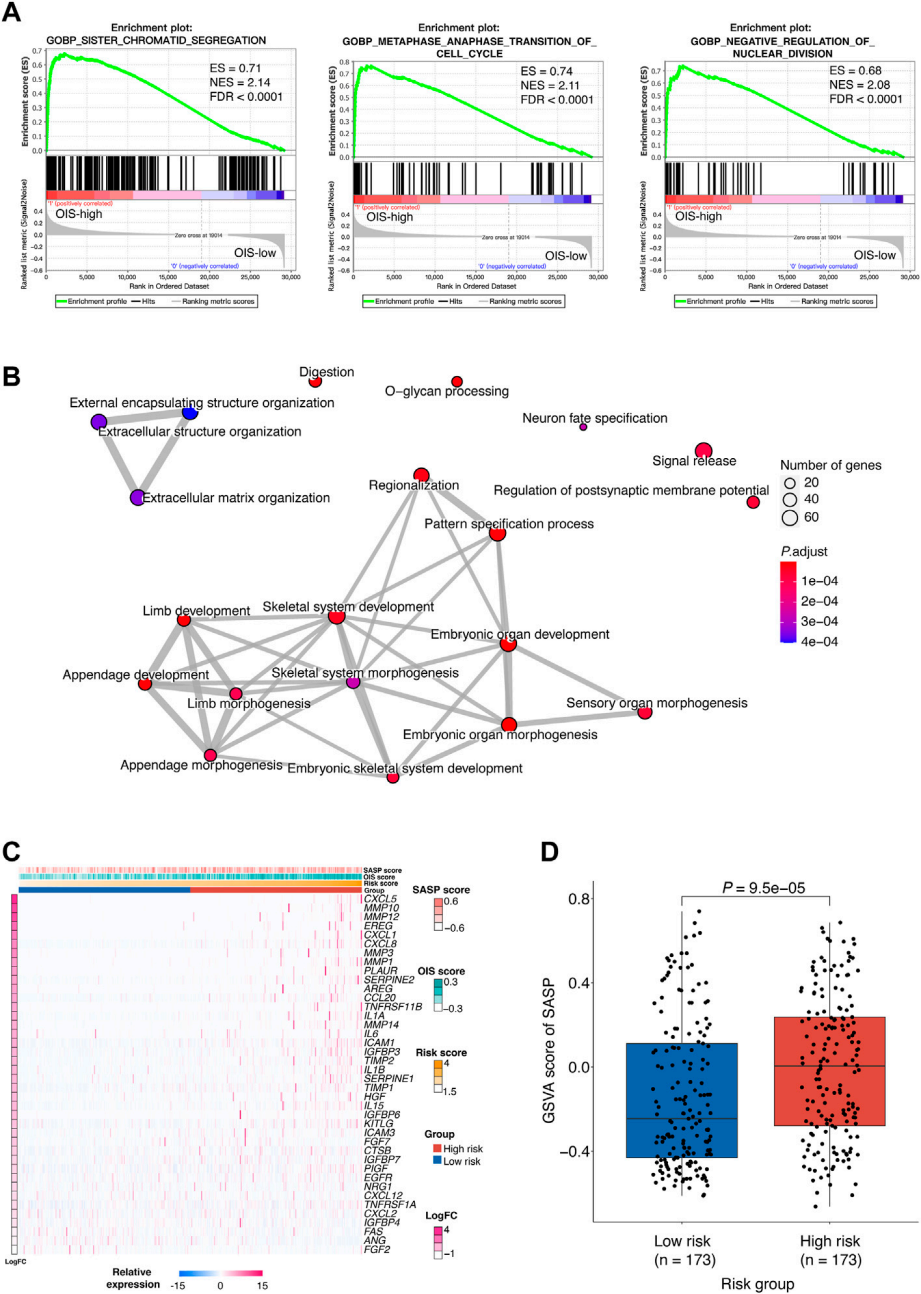
Biological implications of the OIS-related risk score **(A)** GSEA results of the significantly enriched signaling pathways in the HCC patients with high-risk. GSEA was carried out using expression data of TCGA-LIHC tumors. The ESs, NESs and FDRs were determined by GSEA. **(B)** Network diagrams of the enriched signaling pathways with *p* < 0.05. Enrichplot analysis was used to create the network diagrams. The number of genes included in the pathway is represented by the size of the circle, and the number of genes shared by the connected pathways is shown by the thickness of the line. **(C)** A heatmap of the expression levels of SASP-related genes in HCC tumors from TCGA. **(D)** GSVA scores of SASP in the high- and low-risk groups. The *p* value was assessed by Wilcox test. GSEA, gene set enrichment analysis; HCC, hepatocellular carcinoma; TCGA, the cancer genome atlas; ES, enrichment score; NES, normalized enrichment score; FDR, false discovery rate; SASP, senescence-associated secretory phenotype; GSVA, gene set variation analysis.

To investigate the difference between patients with high risk scores and those with low risk scores in the context of biological mechanisms, we employed gene set enrichment analysis (GSEA) in HCCs from TCGA. Based on the risk groups, we found enrichment of sister chromatid segregation, metaphase anaphase transition of the cell cycle and regulation of nuclear division in the high-risk group, indicating abnormal signal of cell proliferation (Figure 5A). We also noticed that pathways such as fatty acid catabolic process, monooxtgenase activity and microbody were enriched in the low-risk group, indicating the preservation of normal liver function (Supplementary Figure S1A). We then performed differential gene expression analysis by DESeq2, and identified 2,683 differentially expressed genes (DEG, *p* < 0.05, abs [logFoldChange] > 1), including 2,090 up-regulated and 593 down-regulated ones (Supplementary Figure S1B). DEGs were subsequently classified into functional classes using GO and KEGG pathway enrichment analysis (Supplementary Figure S1C). GO analysis consisted of biological process (BP) analysis mainly including positive regulation of the development process and signal release signaling pathway. Meanwhile, many of the identified DEGs were found to be associated with neuroactive ligand-receptor interactions and cytokine-cytokine receptor interaction, according to KEGG pathway analysis. Additionally, the results of the network diagrams constructed by enrichplot analysis were consistent with these findings and highlighted the involvement of developmental processes in OIS, such as embryonic organ development, cell differentiation and extracellular matrix organization (Figure 5B).

Cellular senescence is often accompanied by SASP which functions as a strong amplifier of senescence (Glück et al., 2017) and SASP components can actively participate in tumor development (Coppé et al., 2010). To provide further understanding of the functions altered in different risk groups, the relationship between the risk score and the SASP pattern in HCC was investigated. We observed that plenty of the SASP components previously defined (Coppé et al., 2010) were upregulated in the high-risk group (Figure 5C **)**. Among them, IL-1β, IL-6, and CXCL1 have been reported to be factors of immune suppressive SASP that attract myeloid-derived suppressor cells (MDSCs) to inhibit cytotoxic NK and T cell responses in prostate adenocarcinoma (Garcia et al., 2014; Toso et al., 2014). Besides, as part of the SASP, matrix metalloproteinases (MMPs) such as MMP10, MMP12, and MMP3 can lead to cleavage of NKG2D ligands on the surface of senescent tumor cells that allows them to evade NK cell surveillance (Eggert et al., 2016). Furthermore, a significantly higher GSVA score of the SASP signature was obtained in the high-risk group (*p* < 0.0001) (Figure 5D), which implied tumor-promoting effects of SASP in individuals with high-risk scores.

Together, we demonstrated that the profile of the high-risk group was associated with abnormal developmental processes, which further reflects an oncogenic pattern of SASP in HCC.

### Potential regulatory effects of oncogene-induced senescence on immune microenvironment in hepatocellular carcinoma

As senescent cells with SASP can have great influences on immune microenvironment of tumor and render it to a conducive status to tumor growth and progression (Park et al., 2021), the relationship between SASP and risk score led us to further investigate the impact of OIS on immune microenvironment in HCC.

**FIGURE 6:**
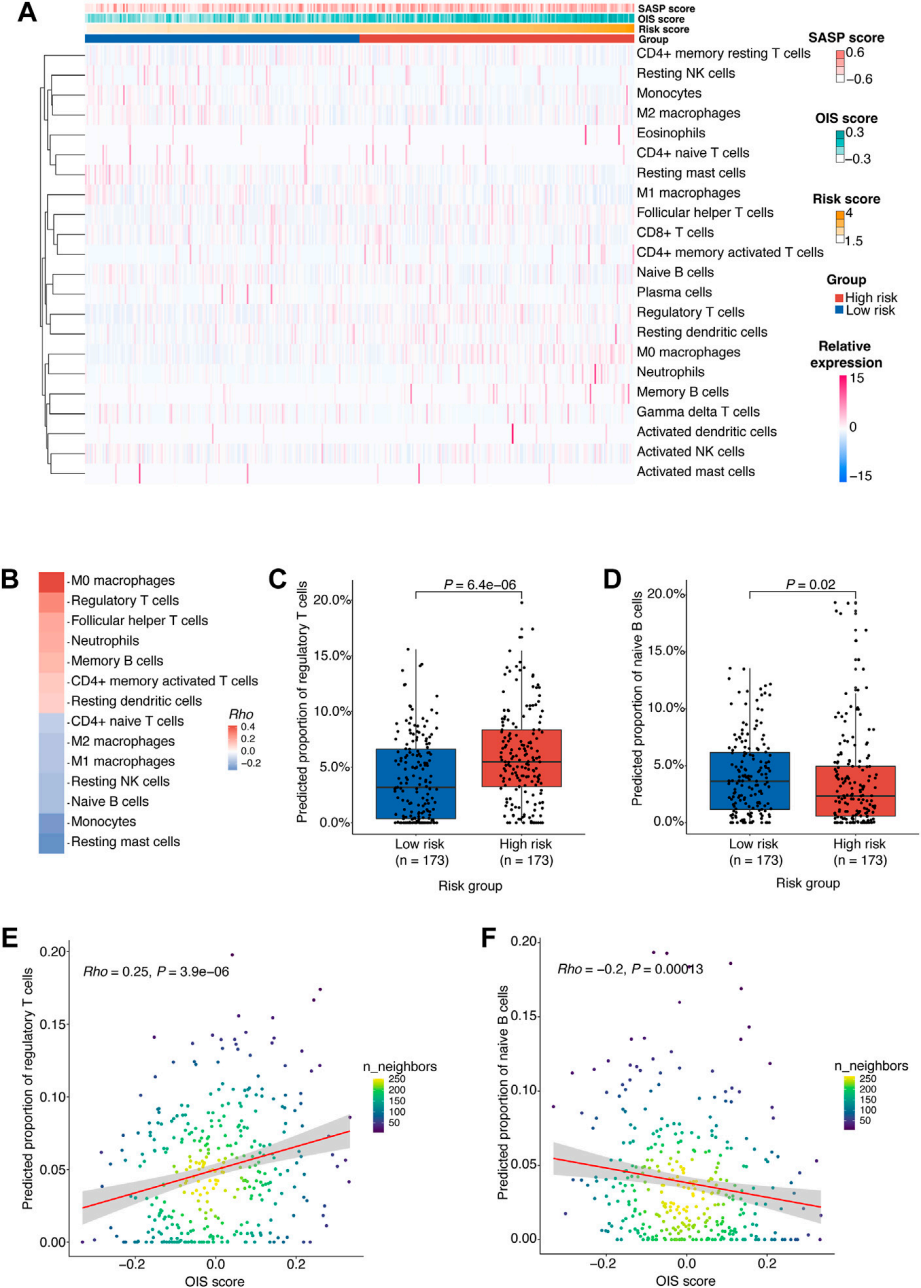
Potential regulatory effects of OIS on the immune microenvironment in HCC.**(A)** 22 types of immune cells’ proportion predicted by CIBERSORT in HCC tumors from TCGA ranked according to the risk score. **(B)** A heatmap of correlations between immune cells proportion and the risk score. The *p* values were determined by Spearman rank correlation analyses and immune cells with *p <* 0.05 were kept. **(C)** The proportions of Tregs in the high- and low-risk groups. **(D)** The proportions of naïve B cells in the high- and low-risk groups. **(E)** A scatter plot of the correlation between the OIS score and the proportion of Tregs. **(F)** A scatter plot of the correlation between the OIS score and the proportion of naïve B cells. In **(C,D)**, the *p* values were assessed by Wilcox test. In **(E,F)**, the correlation coefficients (*rho*) and *p* values were determined by Spearman rank correlation analyses and the color represents the density of the scatters. HCC, hepatocellular carcinoma; TCGA, the cancer genome atlas; OIS, oncogene-induced senescence.

To gain insight into the difference in tumor microenvironment and composition of immune cell infiltrates between different risk groups in HCC, we estimated the relative proportions of 22 immune cell types in HCC tumors from the TCGA-LIHC cohort using CIBERSORT (Newman et al., 2015) (Figure 6A). According to the correlation between the immune cell proportion and the risk score, we found that M0 macrophages, regulatory T cells (Tregs) and follicular helper T cells (Tfhs) were more likely to be enriched in the high-risk group. However, the resting mast cells, monocytes and naïve B cells were more abundant in the low-risk group (Figure 6B).

We next investigated the difference in the proportion of these cells between risk groups (Supplementary Figure S2A) and the correlation between their proportion and the OIS score.

Firstly, we found that the proportion of M0 macrophages is positively correlated with the OIS score (Supplementary Figure S2B). M0 macrophages were identified as non-activated macrophages that can be polarized into two functionally contrasting subtypes: tumor-detrimental M1 and tumor-beneficial M2 phenotypes (Pan et al., 2020). Secondly, the proportion of Tfhs was also found to be positively correlated with the OIS score (Supplementary Figure S2C). Tfhs can impact multiple aspects of the immune system during cancer and usually leads to an effective anti-tumor response (Gu-Trantien et al., 2017). Although many studies have associated Tfhs with survival, there are some instances where Tfhs are reported to be detrimental because of their production of IL-4 (Mayberry et al., 2022). Moreover, we noticed that the proportions of Tregs are higher in the high-risk group (Figure 6C) and have a significant correlation with the OIS score (Figure 6E). Tregs act as a significant subset of CD4^+^ T cells with suppressive effects on a number of immune cells, including natural killer cells, dendritic cells, CD8^+^ T cells and CD4^+^ T cells. Meanwhile, Tregs play an indispensable role in maintaining normal immune homeostasis and peripheral tolerance (Wing et al., 2019). As their suppressive activity in the tumor microenvironment is associated with the loss of anti-tumor immunity (Li et al., 2020), an in-depth understanding of Tregs is essential to the immunotherapy of cancer.

Additionally, we observed a negative correlation between the proportion of monocytes and the OIS score (Supplementary Figure S2D). Monocytes bridge innate and adaptive immune responses and can affect the tumor microenvironment through various mechanisms that induce immune tolerance, angiogenesis, and increased dissemination of tumor cells. Yet monocytes can also give rise to antitumor effectors and activate antigen-presenting cells (Ugel et al., 2021). Meanwhile, the proportion of resting mast cells was also found to be negatively correlated with the OIS score (Supplementary Figure S2C). Resting mast cells can contribute to tissue homeostasis by constantly sampling the microenvironment due to their distinct developmental, phenotypic, and functional plasticity (Frossi et al., 2017). However, to date, the role of mast cells in tumors has been largely ignored, particularly due to the contradictory evidence of a causal relationship between mast cell infiltrates and tumor progression (Maciel et al., 2015). Besides, patients with low-risk score had a significantly higher percentage of naïve B cells (Figure 6D) and there was a negative correlation between its proportion and the OIS score (Figure 6F). Recent data has strongly indicated a critical role for naïve B cells in anti-tumor immunity as their activation can lead to antigen-specific immune memory, which can then differentiate into memory B and plasma cells within the germinal centers (Downs-Canner et al., 2022).

Therefore, it can be reasonably assumed that the OIS of HCC may exert a pivotal role in the regulation of the immune microenvironment, especially promoting effects on Treg cell infiltration and inhibitory effects on naïve B cell infiltration.

### Over-expression of hypomethylated oncogene-induced senescence-related lncRNA NRAV is associated with poor prognosis of hepatocellular carcinoma patients

**FIGURE 7:**
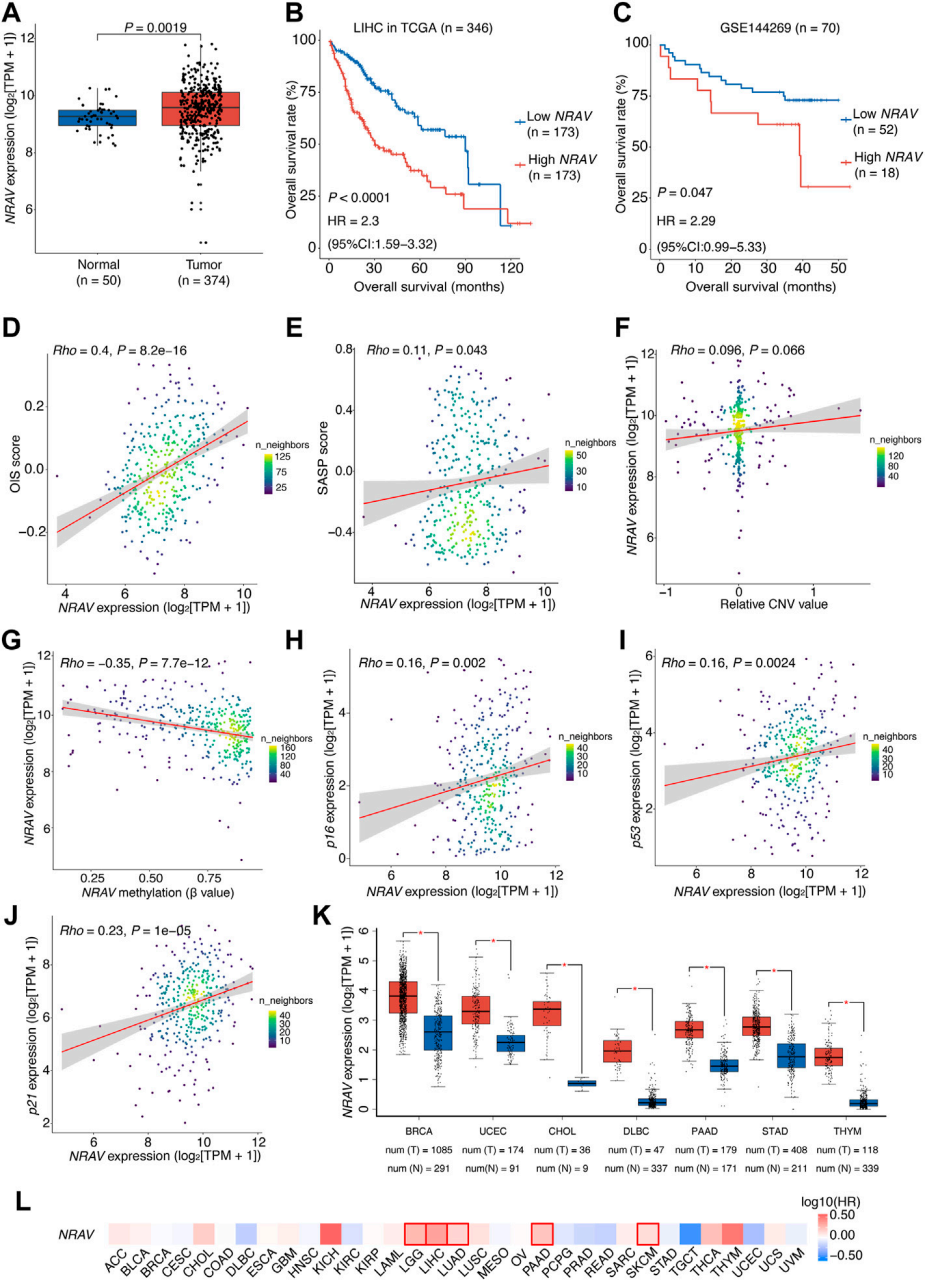
Over-expression of *NRAV* driven mainly by DNA hypomethylation is associated with poor prognosis of HCC patients.**(A)** *NRAV* expression levels in the high- and low-risk groups. Wilcox test was used to get the *p* value. **(B)** Kaplan-Meier survival plots of HCC patients (TCGA-LIHC) grouped by the expression of *NRAV* (stratified by the median expression). **(C)** Kaplan-Meier survival plots of HCC patients (GSE144269) grouped by the expression of *NRAV* (stratified by the upper quartile expression). **(D)** A scatter plot of the correlation between *NRAV* expression and the OIS score. **(E)** A scatter plot of the correlation between *NRAV* expression and the SASP score. **(F)** A scatter plot of the correlation between the relative CNV value of *NRAV* and its expression. **(G)** A scatter plot of the correlation between the DNA methylation level of *NRAV* and its expression. **(H–J)** Scatter plots of the correlations between expression of *NRAV* and senescence-associated markers, including *p16*, *p21* and *p53*. **(K)** Expression levels of *NRAV* in tumors and adjacent non-tumor tissues across different cancer types. The data was collected from GEPIA. (**L**) Survival analyses of *NRAV* expression in TCGA pan-cancer cohorts. The HRs and *p* values were calculated using univariate Cox proportional hazards regression analysis. In **(B,C)**, The *p* value was determined by log-rank test, and the HR with 95% CIs were obtained from multivariate Cox proportional hazards regression analysis. In **(D–J)**, the correlation coefficients (*rho*) and *p* values were calculated using Spearman rank correlation analysis and the color represents the density of the scatters. HCC, hepatocellular carcinoma; HR, hazards ratio; CI, confidence interval; CNV, copy number variation; TCGA, the cancer genome atlas.

Among the 11 OIS-related signature lncRNAs in the prognostic model, *NRAV* exhibited the most significant association with prognosis (LASSO coefficients λ = 0.1483) and can be regarded as an independent risk indicator in HCC according to multivariate Cox proportional hazards regression analysis (*p* < 0.001, HR [95% CI] = 2.28 [1.56–3.31]; Figure 2C). We also observed that *NRAV* had a significantly higher expression level in HCC tissues when compared to the paired non-tumor tissues (Figure 7A) and its higher expression was correlated with a poor OS rate in both TCGA-LIHC and independent HCC cohort (GSE144269) (Figures 7B,C). Besides, the expression of *NRAV* is highly associated with the OIS score (*rho* = 0.4, *p* < 0.0001; Figure 7D) and correlated with the SASP score (*rho* = 0.11, *p* = 0.043; Figure 7E). A previous study has shown that *NRAV* can enhance proliferation and invasion of HCC cells by promoting the Wnt/β-catenin signaling pathway (Wang Q. et al., 2022). Another study showed that *NRAV* is part of an immune-related lncRNA signature acting as a prognostic biomarker for human endometrial cancer (Wang Z. et al., 2021). However, there has been a lack of study on the relationship between *NRAV* and OIS up to now. Thus, we were prompted to assess the role of *NRAV* in HCC OIS.

To this end, we explored the underlying mechanisms that cause the over-expression of *NRAV* in HCC tumors. Through genomic analysis of *NRAV* in HCC tissues based on the dataset from the TCGA cohort, we observed no significant relationship between *NRAV* expression and its genomic copy number (*rho* = 0.096, *p* = 0.066; Figure 7F). Thus, the methylation status of the *NRAV* promoter region was then examined, and we found that the methylation levels were negatively correlated with *NRAV* expression levels (*rho* = -0.35, *p* < 0.0001), suggesting that hypomethylation may contribute to the over-expression of *NRAV* (Figure 7G). Of note, a positive correlation was observed between the expression levels of *NRAV* and the cellular senescence-associated markers (Campisi and d'Adda di Fagagna, 2007), including *p16*, *p21,* and *p53* (Figures 7H–J).

We further assessed the dysregulation and prognostic significance of *NRAV* in pan-cancer. The result showed that there were 7 types of cancer with *NRAV* over-expression in tumor tissues compared to non-tumor tissues, including breast invasive carcinoma (BRCA), uterine corpus endometrial carcinoma (UCEC), cholangiocarcinoma (CHOL), DBLC, pancreatic adenocarcinoma (PAAD), STAD and thymoma (THYM) (Figure 7K). Additionally, *NRAV* also presents a risk-indicative value for LGG, LUAD, PAAD and skin cutaneous melanoma (SKCM) (HR > 1, P < 0.05; Figure 7L) according to pan-cancer survival analyses, suggesting a trans-cancer prognostic value of *NRAV*. Taken together, these results indicated that over-expressed *NRAV* driven by hypomethylation is a risk factor for HCC prognosis, which may act as a modulator of cellular senescence.

## Discussion

A key problem for improving the clinical outcomes of HCC patients is the lack of useful and accurate predictive biomarkers or models. The purpose of this study was to investigate and assess the predictive significance of OIS-related lncRNAs for HCCs. First, we developed and validated a novel 11-lncRNA prognostic risk score model, which served as an independent prognostic factor for HCC patients. Secondly, our findings implied a potential mechanism in the regulation of SASP, which is in charge of the communication between cells undergoing OIS and the tumor immune microenvironment. Thirdly, we identified *NRAV* as a representative hypomethylated OIS-related lncRNA, which is associated with a poor outcome for HCC patients.

Our study is, to our knowledge, the first report to systematically assess the OIS signature in pan-cancer multi-omics data. We identified 10 TCGA tumor types (including STAD, COAD, ACC, UVM, MESO, KIRC, LIHC, LUAD, CESC, and HNSC) that exhibit an OIS-associated outcome. Of note, all of them are limited to solid tumors, for which the OIS signature can be more informative, implying that there may be a divergence of OIS-induced consequences between solid tumors and hematologic malignancies. In addition, we also noticed that the OIS score predicts a decreased risk of death in STAD and COAD, which often exhibit microsatellite instability (MSI) due to deficient DNA mismatch repair mechanisms (dMMR) (Li et al., 2021; Puliga et al., 2021). Current evidence indicates that genomic instability is one of the hallmarks of cellular senescence (López-Otín et al., 2013). Meanwhile, previous studies have also shown that the percentage of MSI in both STAD and COAD increased gradually with increasing age (Polom et al., 2017) and was associated with better prognosis in later onset cohorts (Farrington et al., 2002). Nevertheless, the mechanistic basis for the linkage between MSI genetic status and senescence remains unknown. Our results provide additional justification for more research in this area of senescence.

Human transcriptome sequencing has found tens of thousands of lncRNAs. Increasing research has discovered an increasing number of cancer-related lnRNAs. Some of them play essential roles in tumorigenesis and progression of HCC (Klingenberg et al., 2017), such as *MCM3AP-AS1* (Wang et al., 2019) and *PSTAR* (Qin et al., 2020), which can predict the prognosis of HCC patients. However, it is unfortunate that few senescence-related lncRNAs have been identified. Our study, thus, fills an essential gap in our knowledge of the developmental role of this component of HCC, and the OIS-related lncRNA-based prognostic model outperforms the traditional clinical factors. Besides, our prognostic model is more accurate in predicting HCC patients’ 1-, 3-, and 5-year survival rates (AUC = 0.806, 0.722, and 0.761, respectively) and higher than the previously published models that used 4 glycolysis-related lncRNAs (AUC = 0.747, 0.660, and 0.656, respectively) (Bai et al., 2021), and 5 exosome-related lncRNAs (AUC = 0.63, 0.58, and 0.65, respectively) (Hou et al., 2018). Collectively, our model exhibited excellent short- and long-term prognostic values in HCC patients.

As one of the most distinctive features of senescence, SASP has attracted considerable attention in senescence research because of its arguable contribution to the immune microenvironment (Coppé et al., 2008). On the one hand, senescent cancer cells with SASP can arrest neighboring cancer cells, improve the vasculature for drug delivery and recruit immune cells that can contribute further to tumor suppression (Faget et al., 2019). On the other hand, SASP can promote angiogenesis to advance tumor growth and an epithelial to mesenchymal transition in neighboring cancer cells, which can promote metastasis (Ruhland et al., 2016). This shift in function during senescence, which has not yet been fully clarified, is likely to have significant biological, diagnostic, and therapeutic implications. Thus, it will be crucial to better understand how senescent cancer cells-associated SASP impacts the immune microenvironment. In this study, we characterized the functional linkage between the OIS signature and SASP in HCC. Recent advances in senescence have shed light on the broad role of senescence in regulating tumor-immune microenvironments (Chibaya et al., 2022). Integrated analyses have connected prognosis, immunogenic characteristics and cellular senescence in lung adenocarcinoma (Lin et al., 2021). Immune-suppressive immune cells, such as Tregs and MDSCs, are recruited by the tumor cells by secreting anti-inflammatory cytokines and other chemokines, which inhibit NK and CD8^+^ T cell cytotoxicity and sedate anti-tumor immunity (Shalapour and Karin, 2015). Our results revealed the potential impact of OIS on immune microenvironment, especially promoting effects on Treg cell infiltration. Although the results are speculative at this stage, they may provide insight into lncRNAs’ roles in the regulation of SASP and subsequent immune minienvironment.

The majority of the OIS-associated lncRNAs identified in our risk model are novel lncRNAs without comprehensive annotation and functional relevance, while some ones have been explored to some extent. For instance, emerging evidence shows that *SNHG3* is a novel oncogenic lncRNA that is abnormally expressed in various types of tumor, including osteosarcoma, liver cancer and lung cancer (Xu et al., 2020). High *MIR100HG* expression was also positively associated with clinical stage, tumor invasion, lymph node metastasis, and distant metastasis in gastric cancer (Li et al., 2019). More importantly, *MIR100HG* is functionally significant in establishing senescent phenotype of adult adipose-derived stem cells (Lopez et al., 2017) and its introns including *miR-100*, *miR-125b,* and *let-7a* were all reported to regulate the pace of development or senescence-related degenerative phenotype (Keane and de Magalhães, 2013; Nyholm et al., 2014; Li et al., 2015). *NRAV* has been identified as an immune-related lncRNA in several studies across cancer types, including HCC (Zhou et al., 2021), endometrial cancer (Wang Z. et al., 2021) and lower-grade glioma (Maimaiti et al., 2021). It is also a key regulator of antiviral innate immunity because of its crucial role in the initial transcription of multiple critical interferon-stimulated genes (Ouyang et al., 2014). In terms of mechanism, *NRAV* could influence the modulation of the *miR-199a-3p/CISD2* axis and trigger the Wnt/β-catenin signaling (Wang Q. et al., 2022) which is related to senescence associated stemness (Milanovic et al., 2018). Senescence associated stemness may result in a highly aggressive tumor, driven by Wnt pathway activation independent of the Wnt ligand *via* the SASP and is found to be enriched in relapsed tumors (Wang L. et al., 2022). These findings provide us with clues for future research, whereas the detailed mechanism of how they operate in senescence requires more investigation.

The study also has certain limitations that must be acknowledged. First, our prediction model was developed using TCGA data from the United States while validated using GEO data from Mongolia. Thus, there may be racial differences and HCC etiology differences in this study, and prospective studies in different populations are required for further consideration. Secondly, the lack of specific and reproducible markers of cellular senescence *in vivo* is always the limitation to developing a consensus framework on the role of senescence in cancer biology and tumor immunology (Chibaya et al., 2022). It is unknown whether the OIS-related lncRNAs in our model are capable of distinguishing senescent cells from other cell states related to cell cycle withdrawal, including quiescence, post-mitotic terminal differentiation, and dormancy. Thirdly, the function of many lncRNAs in our model has not been clearly elucidated. It will also be difficult to connect them with the comprehensive networks of senescence transcriptional regulation.

In conclusion, our study demonstrated that the risk score model based on OIS-related lncRNAs expression levels can effectively categorize HCC patients into favorable and unfavorable groups, thereby extending prognostic significance to the traditional clinicopathological risk factors. Furthermore, our risk score model might offer a more convenient and reliable strategy for predicting the prognosis of HCC patients. It can therefore provide critical information for patient prognosis and assist in the selection of suitable disease management strategies. A similar strategy might be utilized to establish other cancer-specific prognostic prediction models.

## Data Availability

Publicly available datasets were analyzed in this study. The names of the repository/repositories and accession numbers can be found in the article/Supplementary Material.
