# Supplementary material for: Identification of senescence-associated long non-coding RNAs to predict prognosis and immune microenvironment in patients with hepatocellular carcinoma
Source: Front Genet. 2022 Oct 13;13:956094. doi: 10.3389/fgene.2022.956094 (PMC9624069; doi:10.3389/fgene.2022.956094)
Supplement: Supplementary file 1 [file DataSheet1.docx]

**Supplementary Materials for：**

**Identification of Senescence-associated Long Non-coding RNAs to Predict Prognosis and Immune Microenvironment in Patients with Hepatocellular Carcinoma**

**Supplementary Figure:**

**
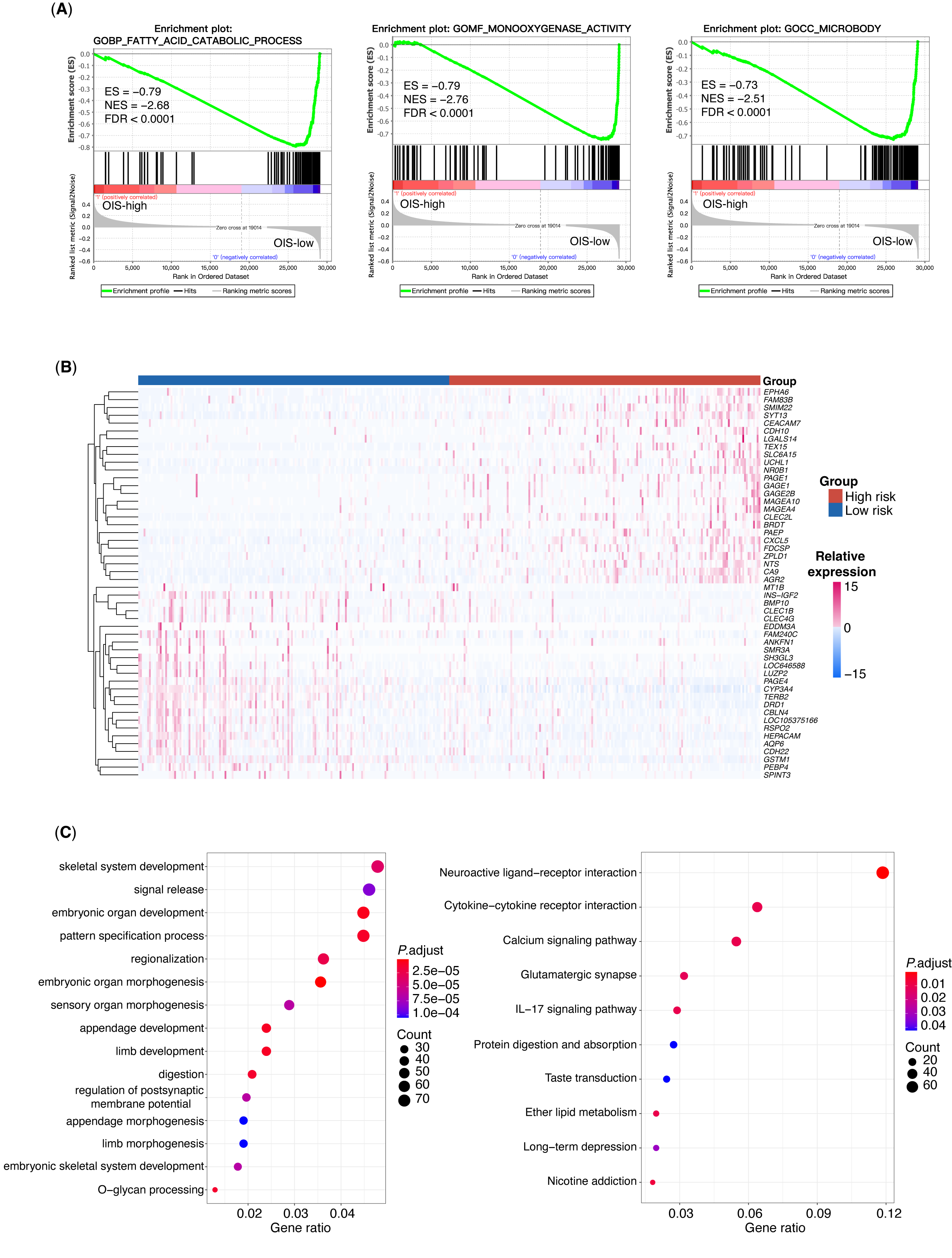
**

**Figure S1. Differentially expressed genes between the high- and low-risk groups and enriched pathways identified by GSEA in the low-risk group.**

(**A**) GSEA results showing the significant enrichment of signaling pathways in the low-risk HCC patients. GSEA was performed based on the expression data of HCC tumors from TCGA. The ESs, NESs and FDRs were determined by GSEA. (**B**) A heatmap of the top 50 differentially expressed genes between the high- and low-risk groups. (**C**) Top significantly enriched GO (left) and KEGG (right) terms in the high-risk group. GSEA, gene set enrichment analysis; HCC, hepatocellular carcinoma; TCGA, the cancer genome atlas; ES, enrichment score; NES, normalized enrichment score; FDR, false discovery rate; GO, gene ontology; KEGG, kyoto encyclopedia of genes and genomes.


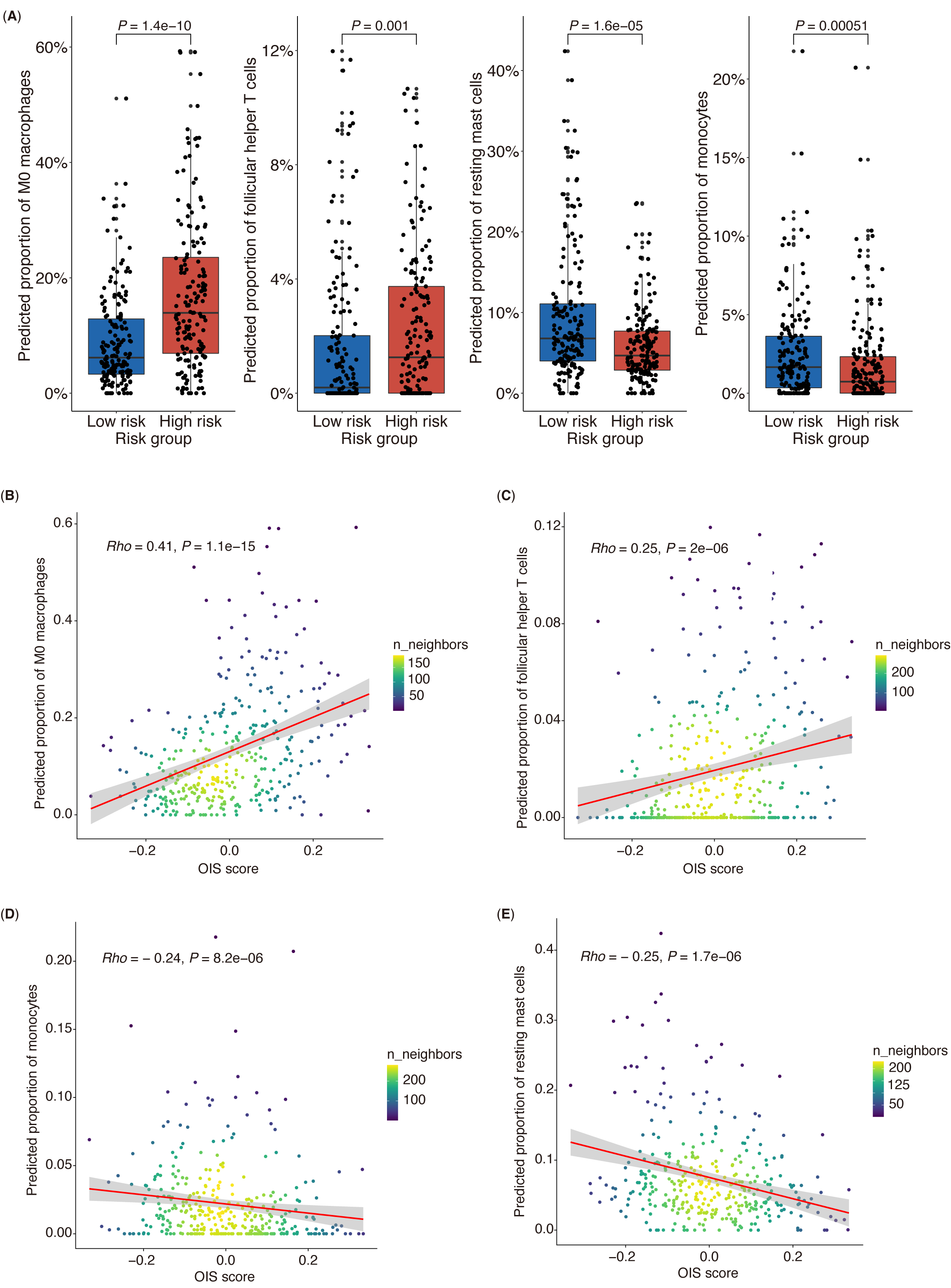


**Figure S2. Correlation between the OIS score and the immune cell proportions predicted by CIBERSORT in HCC tumors from TCGA.**

(**A**) The proportions of M0 macrophages, follicular helper T cells, resting mast cells and monocytes in the high- and low-risk groups. (**B**) A scatter plot of the correlation between the OIS score and the proportion of M0 macrophage cells. (**C**) A scatter plot of the correlation between the OIS score and the proportion of follicular helper T cells. (**D**) A scatter plot of the correlation between the OIS score and the proportion of monocytes. (**E**) A scatter plot of the correlation between the OIS score and the proportion of resting mast cells. In (**A**), the *P* values were assessed by Wilcox test. In (**B**-**E**), the correlation coefficients (*rho*) and *P* values were determined by Spearman rank correlation analyses and the color represents the density of the scatters. HCC, hepatocellular carcinoma; OIS, oncogene-induced senescence.

**Supplementary Tables:**

**Table S1：**Information of datasets in this study.

| **IDs** | **Datasets** | **No. of samples** | **Data types** | **Application** | **Source countries** | **Platforms** |
| --- | --- | --- | --- | --- | --- | --- |
| 1 | TCGA-LIHC | 374 HCC tumor tissues and 50 adjacent non-tumor tissues | mRNA expression | Training cohort: model training | The United States | Illumina HiSeq 2000 and Illumina HumanMethylation450 BeadChip |
| 2 | GSE144269 | 70 paired HCC tumor tissues and adjacent non-tumor tissues | mRNA expression | validation cohort: model validating | Mongolia | Illumina NovaSeq 6000 (Homo sapiens) |

TCGA-LIHC data was downloaded from the TCGA Pan-Cancer Atlas (https://gdc.cancer.gov/about-data/publications/pancanatlas). GSE144269 data was downloaded from the Gene Expression Omnibus (https://www.ncbi.nlm.nih.gov/geo/query/acc.cgi?acc=GSE144269)

**Table S2：**The list of OIS-related lncRNAs.

| **IDs** | **Gene symbols** | **Correlation between the OIS score and lncRNA expression** | |
| --- | --- | --- | --- |
|  |  | ***Rho*** | ***P*** |
| 1 | *AL162412.1* | -0.50 | < 0.01 |
| 2 | *MIR100HG* | -0.47 | < 0.01 |
| 3 | *AL161668.4* | -0.47 | < 0.01 |
| 4 | *LINC02754* | -0.46 | < 0.01 |
| 5 | *TMEM220-AS1* | -0.44 | < 0.01 |
| 6 | *AC021491.4* | -0.44 | < 0.01 |
| 7 | *NAV2-AS4* | -0.43 | < 0.01 |
| 8 | *AP001528.2* | -0.43 | < 0.01 |
| 9 | *AC008537.3* | -0.43 | < 0.01 |
| 10 | *AC137056.2* | -0.42 | < 0.01 |
| 11 | *AC020978.4* | -0.42 | < 0.01 |
| 12 | *LINC02499* | -0.41 | < 0.01 |
| 13 | *AC015908.3* | -0.40 | < 0.01 |
| 14 | *AC004160.2* | -0.40 | < 0.01 |
| 15 | *NRAV* | 0.40 | < 0.01 |
| 16 | *AC131009.3* | 0.40 | < 0.01 |
| 17 | *AC089999.2* | 0.40 | < 0.01 |
| 18 | *NDUFB2-AS1* | 0.40 | < 0.01 |
| 19 | *AL139125.1* | 0.40 | < 0.01 |
| 20 | *CASC19* | 0.40 | < 0.01 |
| 21 | *PRKAR1B-AS1* | 0.41 | < 0.01 |
| 22 | *LINC01138* | 0.41 | < 0.01 |
| 23 | *AC145423.2* | 0.41 | < 0.01 |
| 24 | *MAFG-DT* | 0.41 | < 0.01 |
| 25 | *HDAC2-AS2* | 0.41 | < 0.01 |
| 26 | *AL049840.6* | 0.41 | < 0.01 |
| 27 | *AC009686.2* | 0.41 | < 0.01 |
| 28 | *AC010973.2* | 0.41 | < 0.01 |
| 29 | *AL035071.1* | 0.41 | < 0.01 |
| 30 | *AL355987.4* | 0.41 | < 0.01 |
| 31 | *PVT1* | 0.41 | < 0.01 |
| 32 | *AC002398.1* | 0.42 | < 0.01 |
| 33 | *AC012073.1* | 0.42 | < 0.01 |
| 34 | *AC061992.2* | 0.42 | < 0.01 |
| 35 | *AL365203.2* | 0.42 | < 0.01 |
| 36 | *PELATON* | 0.42 | < 0.01 |
| 37 | *LINC02577* | 0.42 | < 0.01 |
| 38 | *AC026356.1* | 0.42 | < 0.01 |
| 39 | *AC016876.3* | 0.42 | < 0.01 |
| 40 | *AL117335.1* | 0.42 | < 0.01 |
| 41 | *AC023043.4* | 0.42 | < 0.01 |
| 42 | *AP003469.2* | 0.42 | < 0.01 |
| 43 | *ITGB1-DT* | 0.43 | < 0.01 |
| 44 | *ZFAS1* | 0.43 | < 0.01 |
| 45 | *AC009005.1* | 0.43 | < 0.01 |
| 46 | *LINC01503* | 0.43 | < 0.01 |
| 47 | *AC109322.1* | 0.43 | < 0.01 |
| 48 | *AC090192.2* | 0.44 | < 0.01 |
| 49 | *SNHG12* | 0.44 | < 0.01 |
| 50 | *SNHG17* | 0.44 | < 0.01 |
| 51 | *AC135050.1* | 0.44 | < 0.01 |
| 52 | *AC010327.6* | 0.44 | < 0.01 |
| 53 | *AC008622.2* | 0.44 | < 0.01 |
| 54 | *LINC00239* | 0.44 | < 0.01 |
| 55 | *SNHG6* | 0.44 | < 0.01 |
| 56 | *AL031587.5* | 0.44 | < 0.01 |
| 57 | *AC125437.1* | 0.45 | < 0.01 |
| 58 | *LINC00511* | 0.45 | < 0.01 |
| 59 | *AC015912.3* | 0.45 | < 0.01 |
| 60 | *AC116025.2* | 0.45 | < 0.01 |
| 61 | *PPP1R14B-AS1* | 0.46 | < 0.01 |
| 62 | *AL139423.1* | 0.46 | < 0.01 |
| 63 | *AC079209.1* | 0.46 | < 0.01 |
| 64 | *MAPKAPK5-AS1* | 0.46 | < 0.01 |
| 65 | *AL445524.1* | 0.47 | < 0.01 |
| 66 | *AC022092.1* | 0.47 | < 0.01 |
| 67 | *IER3-AS1* | 0.47 | < 0.01 |
| 68 | *AC012676.1* | 0.49 | < 0.01 |
| 69 | *SNHG3* | 0.50 | < 0.01 |
| 70 | *AC026401.3* | 0.51 | < 0.01 |
| 71 | *AC011511.5* | 0.52 | < 0.01 |
| 72 | *PRR7-AS1* | 0.52 | < 0.01 |
| 73 | *ELF3-AS1* | 0.52 | < 0.01 |
| 74 | *MIR4435-2HG* | 0.53 | < 0.01 |
| 75 | *MYG1-AS1* | 0.53 | < 0.01 |
| 76 | *CYTOR* | 0.59 | < 0.01 |

OIS, oncogene-induced senescence.

**Table S3：**The prognostic values of the OIS-related lncRNAs in HCCs from TCGA.

| **IDs** | **Gene symbols** | **Hazard ratios** | ***P*** | **Significant** |
| --- | --- | --- | --- | --- |
| 1 | *AL162412.1* | 0.83 | 0.31 | FALSE |
| 2 | *MIR100HG* | 0.65 | 0.02 | TRUE |
| 3 | *AL161668.4* | 0.66 | 0.03 | TRUE |
| 4 | *LINC02754* | 0.65 | 0.02 | TRUE |
| 5 | *TMEM220-AS1* | 0.60 | < 0.01 | TRUE |
| 6 | *AC021491.4* | 0.55 | < 0.01 | TRUE |
| 7 | *NAV2-AS4* | 0.47 | < 0.01 | TRUE |
| 8 | *AP001528.2* | 0.61 | < 0.01 | TRUE |
| 9 | *AC008537.3* | 0.87 | 0.44 | FALSE |
| 10 | *AC137056.2* | 0.66 | 0.02 | TRUE |
| 11 | *AC020978.4* | 0.73 | 0.08 | FALSE |
| 12 | *LINC02499* | 0.60 | < 0.01 | TRUE |
| 13 | *AC015908.3* | 0.46 | < 0.01 | TRUE |
| 14 | *AC004160.2* | 0.70 | 0.06 | FALSE |
| 15 | *NRAV* | 2.28 | < 0.01 | TRUE |
| 16 | *AC131009.3* | 1.37 | 0.09 | FALSE |
| 17 | *AC089999.2* | 1.73 | < 0.01 | TRUE |
| 18 | *NDUFB2-AS1* | 1.78 | < 0.01 | TRUE |
| 19 | *AL139125.1* | 1.30 | 0.15 | FALSE |
| 20 | *CASC19* | 1.43 | 0.05 | FALSE |
| 21 | *PRKAR1B-AS1* | 1.23 | 0.25 | FALSE |
| 22 | *LINC01138* | 2.01 | < 0.01 | TRUE |
| 23 | *AC145423.2* | 2.05 | < 0.01 | TRUE |
| 24 | *MAFG-DT* | 1.67 | < 0.01 | TRUE |
| 25 | *HDAC2-AS2* | 1.82 | < 0.01 | TRUE |
| 26 | *AL049840.6* | 1.66 | < 0.01 | TRUE |
| 27 | *AC009686.2* | 1.98 | < 0.01 | TRUE |
| 28 | *AC010973.2* | 1.91 | < 0.01 | TRUE |
| 29 | *AL035071.1* | 1.71 | < 0.01 | TRUE |
| 30 | *AL355987.4* | 1.50 | 0.03 | TRUE |
| 31 | *PVT1* | 1.27 | 0.18 | FALSE |
| 32 | *AC002398.1* | 1.66 | < 0.01 | TRUE |
| 33 | *AC012073.1* | 1.55 | 0.02 | TRUE |
| 34 | *AC061992.2* | 1.82 | < 0.01 | TRUE |
| 35 | *AL365203.2* | 1.77 | < 0.01 | TRUE |
| 36 | *PELATON* | 1.39 | 0.07 | FALSE |
| 37 | *LINC02577* | 1.71 | < 0.01 | TRUE |
| 38 | *AC026356.1* | 2.60 | < 0.01 | TRUE |
| 39 | *AC016876.3* | 1.67 | < 0.01 | TRUE |
| 40 | *AL117335.1* | 1.50 | 0.03 | TRUE |
| 41 | *AC023043.4* | 1.55 | 0.02 | TRUE |
| 42 | *AP003469.2* | 1.89 | < 0.01 | TRUE |
| 43 | *ITGB1-DT* | 2.35 | < 0.01 | TRUE |
| 44 | *ZFAS1* | 1.57 | 0.01 | TRUE |
| 45 | *AC009005.1* | 1.78 | < 0.01 | TRUE |
| 46 | *LINC01503* | 1.83 | < 0.01 | TRUE |
| 47 | *AC109322.1* | 1.58 | 0.01 | TRUE |
| 48 | *AC090192.2* | 2.04 | < 0.01 | TRUE |
| 49 | *SNHG12* | 2.16 | < 0.01 | TRUE |
| 50 | *SNHG17* | 1.94 | < 0.01 | TRUE |
| 51 | *AC135050.1* | 1.22 | 0.28 | FALSE |
| 52 | *AC010327.6* | 1.71 | < 0.01 | TRUE |
| 53 | *AC008622.2* | 2.62 | < 0.01 | TRUE |
| 54 | *LINC00239* | 1.37 | 0.08 | FALSE |
| 55 | *SNHG6* | 1.86 | < 0.01 | TRUE |
| 56 | *AL031587.5* | 1.64 | < 0.01 | TRUE |
| 57 | *AC125437.1* | 2.03 | < 0.01 | TRUE |
| 58 | *LINC00511* | 1.47 | 0.03 | TRUE |
| 59 | *AC015912.3* | 1.38 | 0.08 | FALSE |
| 60 | *AC116025.2* | 1.64 | < 0.01 | TRUE |
| 61 | *PPP1R14B-AS1* | 2.34 | < 0.01 | TRUE |
| 62 | *AL139423.1* | 2.28 | < 0.01 | TRUE |
| 63 | *AC079209.1* | 1.75 | < 0.01 | TRUE |
| 64 | *MAPKAPK5-AS1* | 2.48 | < 0.01 | TRUE |
| 65 | *AL445524.1* | 1.58 | 0.01 | TRUE |
| 66 | *AC022092.1* | 1.31 | 0.13 | FALSE |
| 67 | *IER3-AS1* | 1.65 | < 0.01 | TRUE |
| 68 | *AC012676.1* | 2.03 | < 0.01 | TRUE |
| 69 | *SNHG3* | 1.85 | < 0.01 | TRUE |
| 70 | *AC026401.3* | 1.91 | < 0.01 | TRUE |
| 71 | *AC011511.5* | 1.45 | 0.04 | TRUE |
| 72 | *PRR7-AS1* | 2.29 | < 0.01 | TRUE |
| 73 | *ELF3-AS1* | 2.15 | < 0.01 | TRUE |
| 74 | *MIR4435-2HG* | 2.05 | < 0.01 | TRUE |
| 75 | *MYG1-AS1* | 1.63 | < 0.01 | TRUE |
| 76 | *CYTOR* | 2.09 | < 0.01 | TRUE |

OIS, oncogene-induced senescence; HCC, hepatocellular carcinoma; TCGA, the cancer genome atlas.

**Table S4：**GSEA results based on the risk score in the HCCs from TCGA (top50).

| **IDs** | **Gene sets** | **No. of genes** | **Enrichment scores** | **Normalized enrichment scores** | ***P*** | **FDR** | **Groups** |
| --- | --- | --- | --- | --- | --- | --- | --- |
| 1 | GOBP_MITOTIC_SISTER_CHROMATID_SEGREGATION | 164 | 0.71 | 2.13 | < 1E-5 | < 1E-5 | High risk |
| 2 | GOBP_REGULATION_OF_CHROMOSOME_SEGREGATION | 87 | 0.74 | 2.1 | < 1E-5 | < 1E-5 | High risk |
| 3 | GOBP_METAPHASE_ANAPHASE_TRANSITION_OF_CELL_CYCLE | 64 | 0.76 | 2.09 | < 1E-5 | < 1E-5 | High risk |
| 4 | GOBP_SISTER_CHROMATID_SEGREGATION | 194 | 0.68 | 2.09 | < 1E-5 | < 1E-5 | High risk |
| 5 | GOBP_REGULATION_OF_CHROMOSOME_SEPARATION | 71 | 0.75 | 2.04 | < 1E-5 | < 1E-5 | High risk |
| 6 | GOBP_REGULATION_OF_MITOTIC_SISTER_CHROMATID_SEGREGATION | 45 | 0.78 | 2.02 | < 1E-5 | < 1E-5 | High risk |
| 7 | GOBP_MITOTIC_SPINDLE_ORGANIZATION | 117 | 0.68 | 2.00 | < 1E-5 | < 1E-5 | High risk |
| 8 | GOBP_MICROTUBULE_CYTOSKELETON_ORGANIZATION_INVOLVED_IN_MITOSIS | 142 | 0.68 | 2.00 | < 1E-5 | < 1E-5 | High risk |
| 9 | GOBP_MITOTIC_NUCLEAR_DIVISION | 296 | 0.63 | 1.99 | < 1E-5 | < 1E-5 | High risk |
| 10 | GOCC_CONDENSED_CHROMOSOME_CENTROMERIC_REGION | 117 | 0.68 | 1.98 | < 1E-5 | < 1E-5 | High risk |
| 11 | GOCC_SPINDLE_MIDZONE | 36 | 0.80 | 1.97 | < 1E-5 | 8.87E-05 | High risk |
| 12 | GOBP_CHROMOSOME_SEPARATION | 95 | 0.68 | 1.97 | < 1E-5 | 1.62E-04 | High risk |
| 13 | GOCC_KINETOCHORE | 137 | 0.66 | 1.97 | < 1E-5 | 1.49E-04 | High risk |
| 14 | GOBP_NUCLEAR_CHROMOSOME_SEGREGATION | 268 | 0.62 | 1.97 | < 1E-5 | 1.39E-04 | High risk |
| 15 | GOBP_ATTACHMENT_OF_SPINDLE_MICROTUBULES_TO_KINETOCHORE | 35 | 0.79 | 1.96 | < 1E-5 | 1.95E-04 | High risk |
| 16 | GOBP_CHROMOSOME_SEGREGATION | 330 | 0.61 | 1.96 | < 1E-5 | 1.83E-04 | High risk |
| 17 | GOBP_NEGATIVE_REGULATION_OF_CHROMOSOME_ORGANIZATION | 91 | 0.68 | 1.95 | < 1E-5 | 1.72E-04 | High risk |
| 18 | GOBP_NEGATIVE_REGULATION_OF_NUCLEAR_DIVISION | 54 | 0.74 | 1.95 | < 1E-5 | 1.62E-04 | High risk |
| 19 | GOCC_CHROMOSOME_CENTROMERIC_REGION | 196 | 0.63 | 1.93 | < 1E-5 | 3.08E-04 | High risk |
| 20 | GOBP_MITOTIC_METAPHASE_PLATE_CONGRESSION | 50 | 0.73 | 1.93 | < 1E-5 | 2.92E-04 | High risk |
| 21 | GOBP_REGULATION_OF_MITOTIC_NUCLEAR_DIVISION | 109 | 0.66 | 1.93 | < 1E-5 | 3.71E-04 | High risk |
| 22 | GOBP_NEGATIVE_REGULATION_OF_METAPHASE_ANAPHASE_TRANSITION_OF_CELL_CYCLE | 41 | 0.77 | 1.93 | < 1E-5 | 4.43E-04 | High risk |
| 23 | GOCC_CONDENSED_CHROMOSOME | 215 | 0.62 | 1.93 | < 1E-5 | 4.23E-04 | High risk |
| 24 | GOBP_PROTEIN_LOCALIZATION_TO_CHROMOSOME_CENTROMERIC_REGION | 25 | 0.83 | 1.92 | < 1E-5 | 4.06E-04 | High risk |
| 25 | GOBP_SPINDLE_ORGANIZATION | 181 | 0.63 | 1.92 | < 1E-5 | 4.28E-04 | High risk |
| 26 | GOBP_ORGANIC_ACID_CATABOLIC_PROCESS | 256 | 0.77 | 2.83 | < 1E-5 | < 1E-5 | Low risk |
| 27 | GOBP_MONOCARBOXYLIC_ACID_CATABOLIC_PROCESS | 131 | 0.79 | 2.73 | < 1E-5 | < 1E-5 | Low risk |
| 28 | GOBP_FATTY_ACID_CATABOLIC_PROCESS | 106 | 0.79 | 2.66 | < 1E-5 | < 1E-5 | Low risk |
| 29 | GOBP_CELLULAR_AMINO_ACID_CATABOLIC_PROCESS | 104 | 0.79 | 2.64 | < 1E-5 | < 1E-5 | Low risk |
| 30 | GOBP_PEROXISOME_ORGANIZATION | 83 | 0.79 | 2.56 | < 1E-5 | < 1E-5 | Low risk |
| 31 | GOBP_ALPHA_AMINO_ACID_CATABOLIC_PROCESS | 86 | 0.78 | 2.56 | < 1E-5 | < 1E-5 | Low risk |
| 32 | GOBP_SMALL_MOLECULE_CATABOLIC_PROCESS | 428 | 0.65 | 2.53 | < 1E-5 | < 1E-5 | Low risk |
| 33 | GOMF_MONOOXYGENASE_ACTIVITY | 101 | 0.74 | 2.52 | < 1E-5 | < 1E-5 | Low risk |
| 34 | GOBP_FATTY_ACID_BETA_OXIDATION | 75 | 0.78 | 2.52 | < 1E-5 | < 1E-5 | Low risk |
| 35 | GOBP_PEROXISOMAL_TRANSPORT | 72 | 0.80 | 2.51 | < 1E-5 | < 1E-5 | Low risk |
| 36 | GOCC_MICROBODY | 136 | 0.73 | 2.50 | < 1E-5 | < 1E-5 | Low risk |
| 37 | HP_ABNORMAL_CIRCULATING_AMINO_ACID_CONCENTRATION | 113 | 0.73 | 2.49 | < 1E-5 | < 1E-5 | Low risk |
| 38 | GOBP_ALPHA_AMINO_ACID_METABOLIC_PROCESS | 188 | 0.69 | 2.49 | <1E-5 | < 1E-5 | Low risk |
| 39 | GOCC_MICROBODY_LUMEN | 51 | 0.84 | 2.48 | <1E-5 | < 1E-5 | Low risk |
| 40 | GOCC_MITOCHONDRIAL_MATRIX | 474 | 0.64 | 2.48 | <1E-5 | < 1E-5 | Low risk |
| 41 | GOBP_DRUG_METABOLIC_PROCESS | 45 | 0.83 | 2.46 | <1E-5 | < 1E-5 | Low risk |
| 42 | GOBP_RESPONSE_TO_XENOBIOTIC_STIMULUS | 131 | 0.7 | 2.44 | <1E-5 | < 1E-5 | Low risk |
| 43 | HP_ABNORMAL_CIRCULATING_PROTEINOGENIC_AMINO_ACID_CONCENTRATION | 76 | 0.76 | 2.42 | <1E-5 | < 1E-5 | Low risk |
| 44 | GOBP_LIPID_OXIDATION | 110 | 0.72 | 2.40 | <1E-5 | < 1E-5 | Low risk |
| 45 | GOMF_STEROID_HYDROXYLASE_ACTIVITY | 38 | 0.85 | 2.39 | <1E-5 | < 1E-5 | Low risk |
| 46 | GOBP_CELLULAR_AMINO_ACID_METABOLIC_PROCESS | 328 | 0.64 | 2.39 | <1E-5 | < 1E-5 | Low risk |
| 47 | GOBP_LONG_CHAIN_FATTY_ACID_METABOLIC_PROCESS | 119 | 0.69 | 2.39 | <1E-5 | < 1E-5 | Low risk |
| 48 | GOBP_STEROID_METABOLIC_PROCESS | 325 | 0.63 | 2.39 | <1E-5 | < 1E-5 | Low risk |
| 49 | GOMF_VITAMIN_B6_BINDING | 54 | 0.78 | 2.37 | <1E-5 | < 1E-5 | Low risk |
| 50 | GOMF_OXIDOREDUCTASE_ACTIVITY_ACTING_ON_PAIRED_DONORS_WITH_INCORPORATION_OR_REDUCTION_OF_MOLECULAR_OXYGEN_NAD_P_H_AS_ONE_DONOR_AND_INCORPORATION_OF_ONE_ATOM_OF_OXYGEN | 48 | 0.8 | 2.36 | <1E-5 | < 1E-5 | Low risk |

GSEA, gene set enrichment analysis; HCC, hepatocellular carcinoma; TCGA, the cancer genome atlas; FDR, false discovery rate.
